# Interobserver variability in target volume delineation for CT/MRI simulation and MRI-guided adaptive radiotherapy in rectal cancer

**DOI:** 10.1259/bjr.20210350

**Published:** 2021-11-29

**Authors:** Ingrid White, Arabella Hunt, Thomas Bird, Sarah Settatree, Heba Soliman, Dualta Mcquaid, David Dearnaley, Susan Lalondrelle, Shree Bhide

**Affiliations:** 1 Guys and St Thomas’ NHS Foundation Trust, London, UK; 2 The Joint Department of Physics at the Institute of Cancer Research and the Royal Marsden NHS Foundation Trust, London, UK; 3 University Hospitals Bristol NHS Foundation Trust, Bristol, UK

## Abstract

**Objectives:**

Quantify target volume delineation uncertainty for CT/MRI simulation and MRI-guided adaptive radiotherapy in rectal cancer. Define optimal imaging sequences for target delineation.

**Methods:**

Six experienced radiation oncologists delineated clinical target volumes (CTVs) on CT and 2D and 3D-MRI in three patients with rectal cancer, using consensus contouring guidelines. Tumour GTV (GTVp) was also contoured on MRI acquired week 0 and 3 of radiotherapy. A STAPLE contour was created and volume and interobserver variability metrics were analysed.

**Results:**

There were statistically significant differences in volume between observers for CT and 2D-MRI-defined CTVs (*p* < 0.05). There was no significant difference between observers on 3D-MRI. Significant differences in volume were seen between observers for both 2D and 3D-MRI-defined GTVp at weeks 0 and 3 (*p* < 0.05). Good interobserver agreement (IOA) was seen for CTVs delineated on all imaging modalities with best IOA on 3D-MRI; median Conformity index (CI) 0.74 for CT, 0.75 for 2D-MRI and 0.77 for 3D-MRI. IOA of MRI-defined GTVp week 0 was better compared to CT; CI 0.58 for CT, 0.62 for 2D-MRI and 0.7 for 3D-MRI. MRI-defined GTVp IOA week three was worse compared to week 0.

**Conclusion:**

Delineation on MRI results in smaller volumes and better IOA week 0 compared to CT. 3D-MRI provides the best IOA in CTV and GTVp. MRI-defined GTVp on images acquired week 3 showed worse IOA compared to week 0. This highlights the need for consensus guidelines in GTVp delineation on MRI during treatment course in the context of dose escalation MRI-guided rectal boost studies.

**Advances in knowledge:**

Optimal MRI sequences for CT/MRI simulation and MRI-guided adaptive radiotherapy in rectal cancer have been defined.

## Introduction

Neoadjuvant chemoradiation in rectal cancer increases the rate of a negative circumferential resection margin (CRM) and increases local control.^1,2^ Meta-analysis shows that response to neoadjuvant chemoradiation is dose-dependent with pathological complete response (pCR) rates increasing to 20.4% when doses > 60 Gy are delivered.^2^ There is now a move towards organ preservation in patients who have had a complete response on post-treatment MRI to spare morbidity form surgery.^3^ Increasingly conformal treatments and a shift towards rectal boost mean that accurate target definition is essential.

MRI is the gold-standard imaging modality for diagnosis and staging in rectal cancer.^4^ Compared to CT, it provides superior soft tissue contrast for discrimination between normal tissue structures and between tumour and normal rectum. Improved target localisation with MRI at radiotherapy treatment planning (RTP) will enable more accurate delineation of rectal gross tumour volume (GTVp). MRI at treatment delivery will facilitate smaller planning target volume (PTV) margins and reduced organ at risk (OAR) dose and toxicity.^5^ It will also enable adaptation to clinical target volume (CTV) and GTVp during treatment. Accounting for uncertainty in target volume delineation is particularly important in adaptive radiotherapy (ART) with a view to reducing PTV margins.

MRI-guided radiotherapy technologies now used in clinical practice, integrate MRI with EBRT (external beam radiotherapy) delivery, providing MRI data immediately before and after treatment, and simultaneously with treatment delivery.^6–10^ MRI for RTP has different requirements to diagnostic MRI and specific solutions are required.^5^ MRI must be acquired in 3D in MRI-only simulation and treatment workflows. No studies have evaluated uncertainty in target volume delineation on 3D-MRI or on imaging acquired during treatment course. This study will evaluate interobserver variability (IOV) in target volume delineation on RTP CT and MRI to define optimal imaging sequence for CTV and GTVp delineation. It will quantify IOV on 3D-MRI and evaluate IOV in MRI-defined GTVp at week 3 of treatment for rectal boost ART. This is important for radiation delivery on MRI only platforms such as the MR Linac, and implementation of clinical trials adapting boost dose to an MRI-defined GTV based on target motion and treatment response.

## Methods and materials

### Patients and treatment

Three patients with histological diagnosis of rectal cancer, planned for treatment with radical radiotherapy, were prospectively recruited into a study approved by the local ethics committee. Treatment consisted of CT-based EBRT with 45 Gy in 25 fractions to the mesorectum and elective lymph node (LN) volume and simultaneous integrated rectal boost of 52.5 Gy in 25 fractions to the tumour and nodal GTV, delivered with volumetric arc therapy (VMAT). All patients received concomitant chemotherapy with capecitabine.

### CT and MRI technique

Each patient underwent a RTPCT in supine position with intravenous contrast. Patients drank 700 ml water after complete bladder voiding one hour before the CT. The CT was acquired with a slice thickness of 2.5 mm, in-plane resolution of 0.97 mm pixels and FOV 500 mm. MRI sequences, optimised for RTP, were acquired with a 1.5 T Siemens radiology scanner (Siemens, Erlangen, Germany) on the same day as the CT. These images have been described as being acquired at week 0. Patient position and preparation was as for treatment planning. MRI sequences used for segmentation were a *T*_2_W 2D TSE and *T*_2_W 3D SPACE sequence. MRI sequence parameters are summarised in Table 1. An additional RTPMRI was acquired in each patient during week 3 of radiotherapy treatment.

**Table 1.** Summary of MRI sequence parameters

Sequence	Magnet (T)	TR (ms)	TE (ms)	FOV (mm)	Slice thickness (mm)	Slices	Matrix	Pixel
*T*_2_W 3D SPACE	1.5	1700	95	250 × 250 x 166	0.8	207	320 × 320	0.8 × 0.8
*T*_2_W 2D TSE	1.5	4700	91	170 × 170 x 180	3	60	256 × 256	0.7 × 0.7

2D, two-dimensional; 3D, three-dimensional; FOV, Field of view; T, Tesla; TE, Echo time; TR, Relaxation time; ms, Milliseconds.

### Contouring

**Figure 1.**
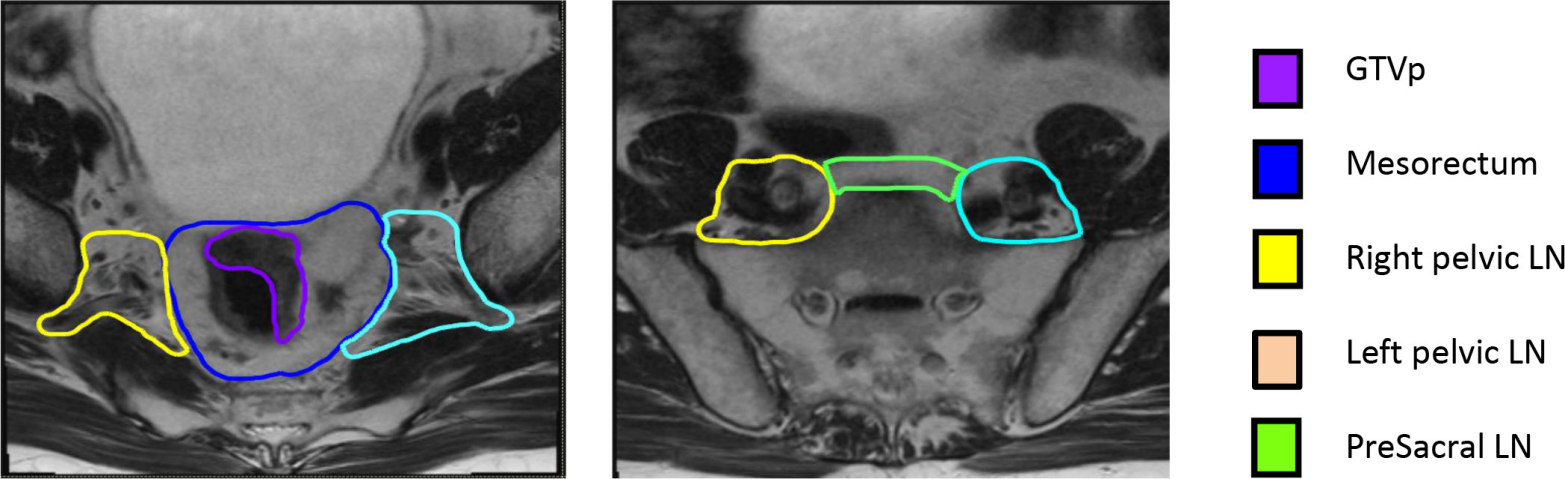
Example contoured structures on 2D-MRI. GTVp, gross tumour volume; LN, lymph node.

Six radiation oncologists at the Royal Marsden NHS Foundation Trust, with experience in treatment of rectal cancer, delineated target volumes in the axial plane in RayStation treatment planning system (RaySearch Laboratories, Stockholm, Sweden). CTV structures were contoured on images acquired at week 0, following target volume delineation guidelines summarised in Table 2, based on international consensus guidelines for delineation in rectal cancer.^11^ Figure 1 provides example-contoured structures on 2D-MRI. CTV was subdivided into separate structures; mesorectum, right pelvic LNs, left pelvic LNs and presacral LNs. GTVp was also delineated. This was defined as visible rectal tumour, excluding normal rectal wall. Window levelling was pre-set, but could be adjusted by observers and observers were blinded to other physician’s contours. The GTVp was also contoured on 2D and 3D-MRI acquired at week 3. A “simultaneous truth and performance estimation” (STAPLE) contour^12^ was created from all six clinician contours for each region of interest (ROI) on each image data set, in each patient to create the “gold-standard” contour.

**Table 2.** Target volume definition contouring guidelines

Target	Border	Definition
Mesorectum	Superior	First bifurcation internal iliac artery
	Inferior	Insertion levator ani muscle
	Lateral	Mesorectal fascia, excluding small bowel
	Anterior	Mesorectal fascia, excluding small bowel
	Posterior	Bone (including pre-sacral fat layer) or pelvic floor muscle, edit out bowel loops, bone, muscle and neuro-foramen
Right and left LN	Superior	Sacral promontory (except superior tumours where superior extension 2 cm above superior extent of GTVp)
	Inferior	Acetabular roof, where pririformis muscle disappears and fascia appears
	Lateral	Pelvic wall muscles/ bone, include whole fat area posterior to the ilium
	Medial	7 mm lateral to iliac vessels/ mesorectal fascia, exclude seminal vesicles, uterus, vagina, small bowel and bladder
	Anterior	7 mm anterior to internal iliac vessels until below level of second bifurcation of internal iliac vessels and then anterior border is up to ½ the obturator muscle
	Posterior	Bone or pelvic floor floor muscles, exclude neuro-foramen
Presacrum LN	Superior	Superior border of right and left LN region
	Inferior	Superior border of mesorectum
	Lateral	Right and left LN regions
	Anterior	1 cm anterior to sacrum
	Posterior	Bone, include pre-sacral fat
GTVp		Visible primary tumour, omitting the lumen and uninvolved rectal wall

GTV, gross tumour volume; LN, lymph node.

### Volumetric analysis

Simple volume analysis and IOV measuring volume and spatial relationship between observer contours were computed. Volumes were calculated per observer, per patient and per modality and were analysed between observers per modality, and between modalities. IOV was calculated for each ROI on each data set by comparing individual observer contours against the STAPLE contour and calculating the average across all observers. The IOV metrics calculated included the conformity index (CI), dice similarity coefficient (DSC), mean and maximum distance to agreement (DTA), geographical miss index (GMI) and discordance index (DI).^13–17^

The CI is the ratio of the common volume to the encompassed volume. It is calculated using A∩B/ AUB with perfect overlap and agreement of contours resulting in a CI = 1.0^13^ . DSC is defined as two times the intersection of the two volumes, divided by the sum of the two volumes; DSC (A,B)=2(A∩B)/(*A* + B).^14^ As for the CI, values closest to 1.0 represent greater agreement. DTA is a geometrical parameter that measures the per voxel shortest distance from the surface of one structure to another, the ideal measure being 0 mm.^15^ The mean DTA measures the average of all these distances. The GMI is the ratio of the gold-standard volume, which does not include the delineated volume, with the gold standard volume; (B not A)/B, where A is the observer delineated volume and B is the gold-standard volume. A value closer to 0 indicates that the gold-standard is completely covered by the observer volume. The greater the GMI, the greater the likelihood of inadequate dose coverage.^16^ The DI measures the ratio of the intersection volume between the delineated volume and the gold-standard, with the delineated volume, which is subtracted from one; DI = 1- (A∩B/A), where *A* = the observer delineated contour and *B* = the gold-standard consensus volume.^17^ A value of 1 represents complete discordance.

### Topographic analysis

Topographic analysis was performed on delineated structures using a script created in RayStation at our institution, Royal Marsden NHS Trust. This script used the graphical capabilities in RayStation plan analysis to create consensus contour agreement maps. Using the dose grid and radiotherapy plan on different image data sets, a dose statistic is allocated to each voxel within observer-contoured volumes. This dose statistic reflects the number of observers that included that voxel within their contour. The 95% isodose and encompassing volume is illustrated in pink. This represents 95% agreement between observers in the volume to be contoured in the delineated structure. Sharp dose fall-off represents high agreement and slow dose fall-off represents increasing variability in contours. The subsequent dose-surface map was used to visually identify the location of areas of poor agreement within 3D CTV and GTVp structures and reasons for these discrepancies were investigated.

### Statistical analysis

Statistical analysis was performed using GraphPad Prism (v. 8.1.2). All continuous numerical variables are presented as median and range values. Variations in volumetric and IOV data were assessed using Friedman’s test with Dunn’s test for multiple comparisons. Values were considered statistically significant if the adjusted *p* value was <0.05.

## Results

### Patient and tumour characteristics

All patients had stage T3N1cM0 cancers with EMVI and threatened or involved CRM. Two patients had mid-rectal tumours and one patient had an upper rectal tumour. Rectal tumour length was 45, 47 and 50 mm. Distance of tumour from the anal verge was 70, 100 and 130 mm respectively. All patients had CT and MRI at week 0 on the same day, and additional MRI at week 3.

### Volume measurements

**Figure 2.**
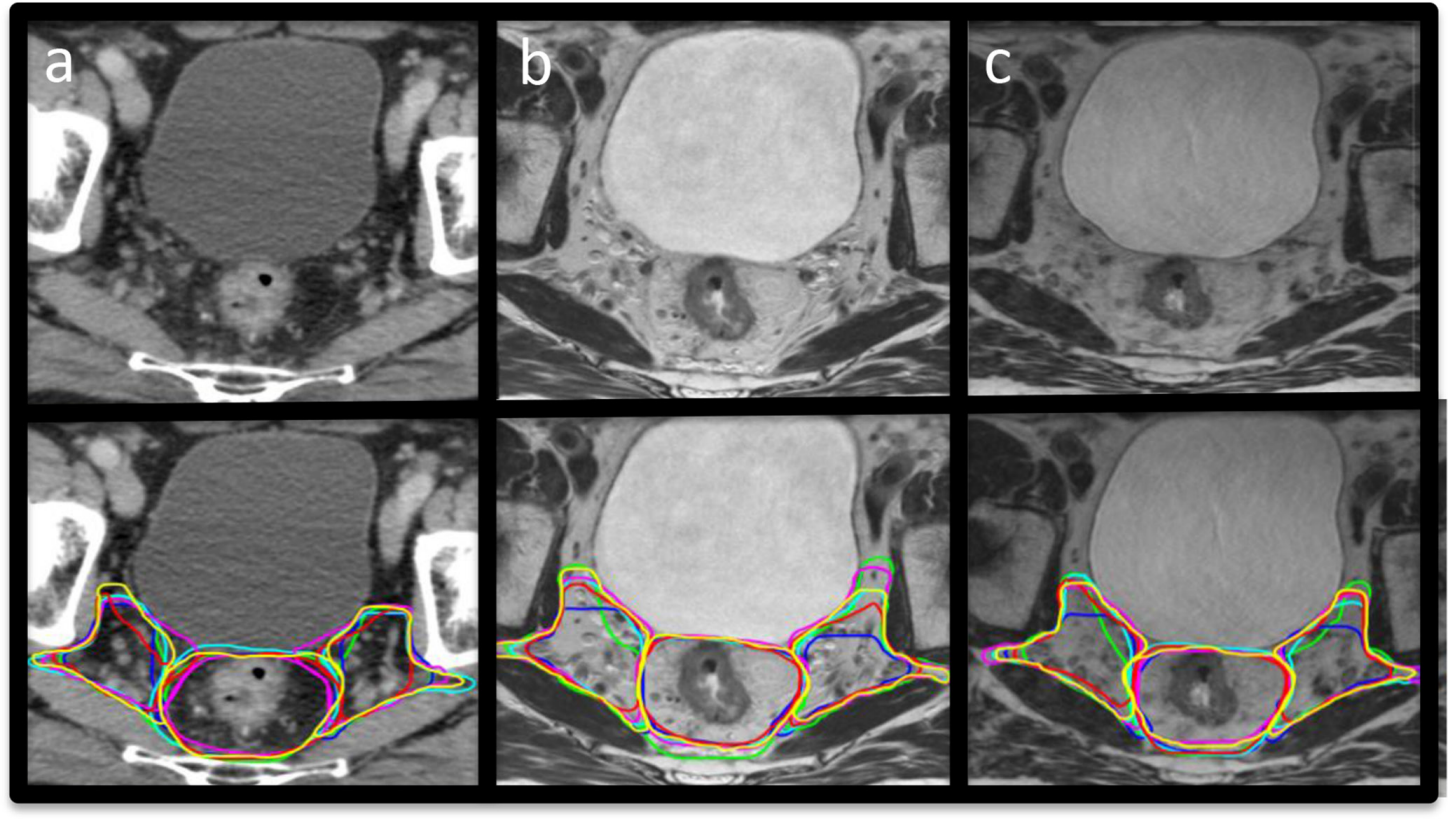
Example images with and without observer CTV contours at week 0. (a) CT, (b) 2D-MRI and (c) 3D-MRI. CTV, clinical target volume.

**Figure 3.**
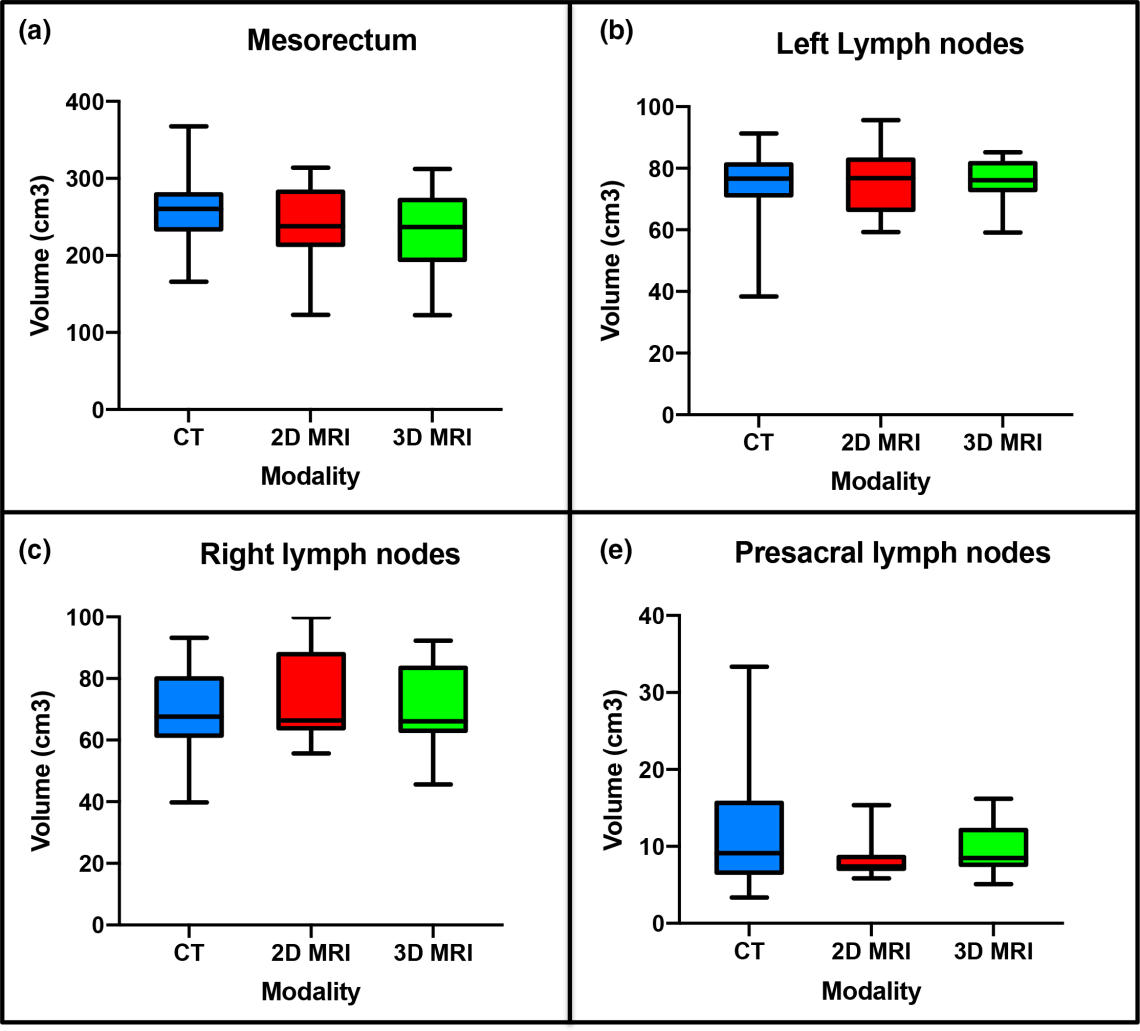
Volumes per modality for all patients combined 1. mesorectum, 2. left pelvic LN volumes, 3. right pelvic LN volumes and 4. presacral LN volumes. LN, lymph node.

Example CTV structure delineations from all six observers on CT, 2D and 3D-MRI week 0 are shown in Figure 2. Volumes for these structures per modality for all patients combined are illustrated in Figure 3. There was a statistically significant difference in volume between observer CTVs when delineated on CT or 2D-MRI (*p* = 0.01 and *p* = 0.04 respectively). There was no significant difference in volume between observer CTV structures delineated on 3D-MRI (*p* = 0.24). The largest variability in volume was seen for the mesorectum, where 2D and 3D-MRI delineated volumes were smaller compared to CT (median volumes; CT 260.6 cm^3^, 2D-MRI 237.7 cm^3^ and 3D-MRI 236.7 cm^3^). All contours were considered to be compliant with the protocol.

**Figure 4.**
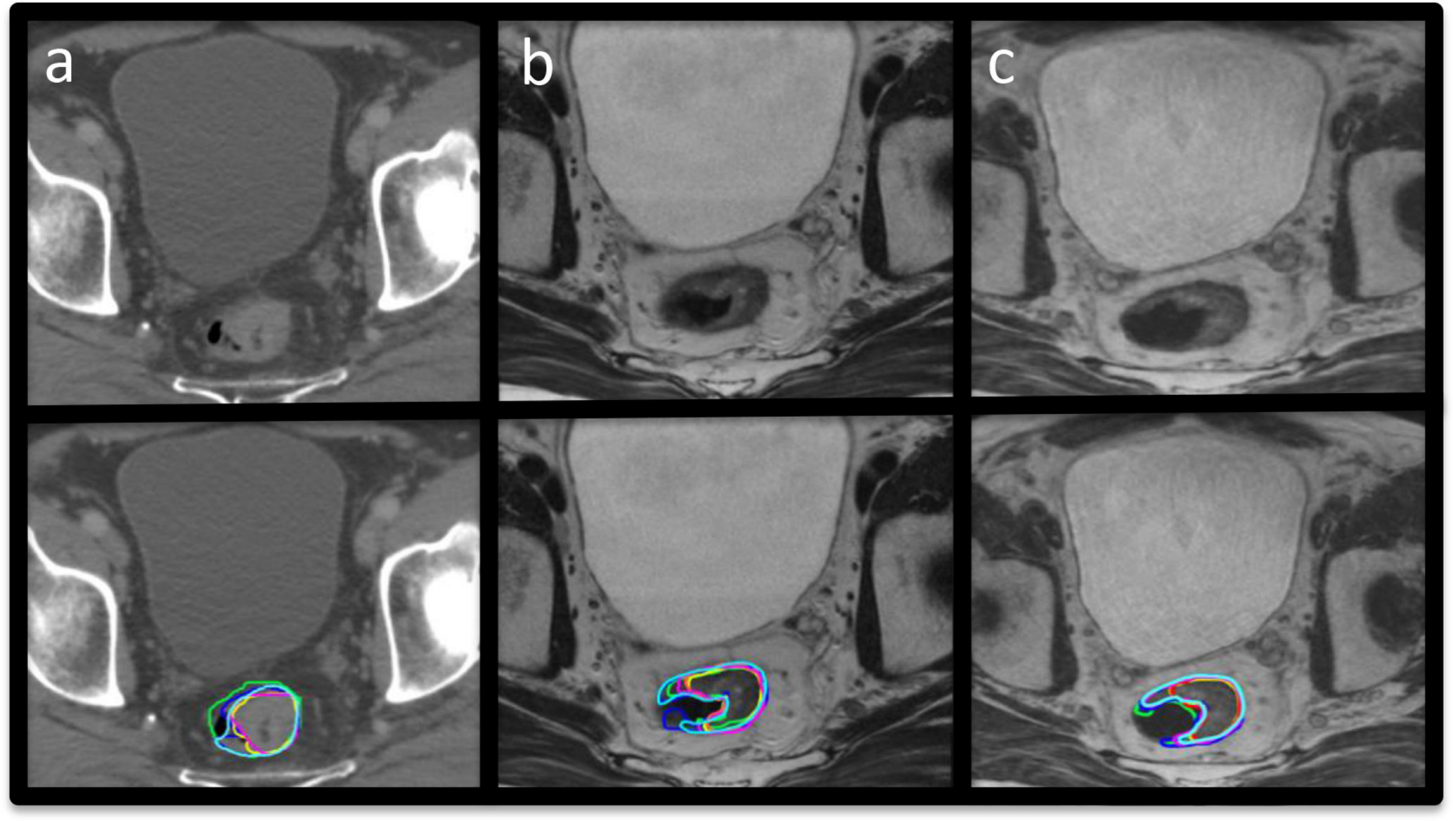
Example images with and without observer GTVp contours at week 0. (a) CT, (b) 2D-MRI and (c) 3D-MRI. GTVp, gross tumour volume.

**Figure 5.**
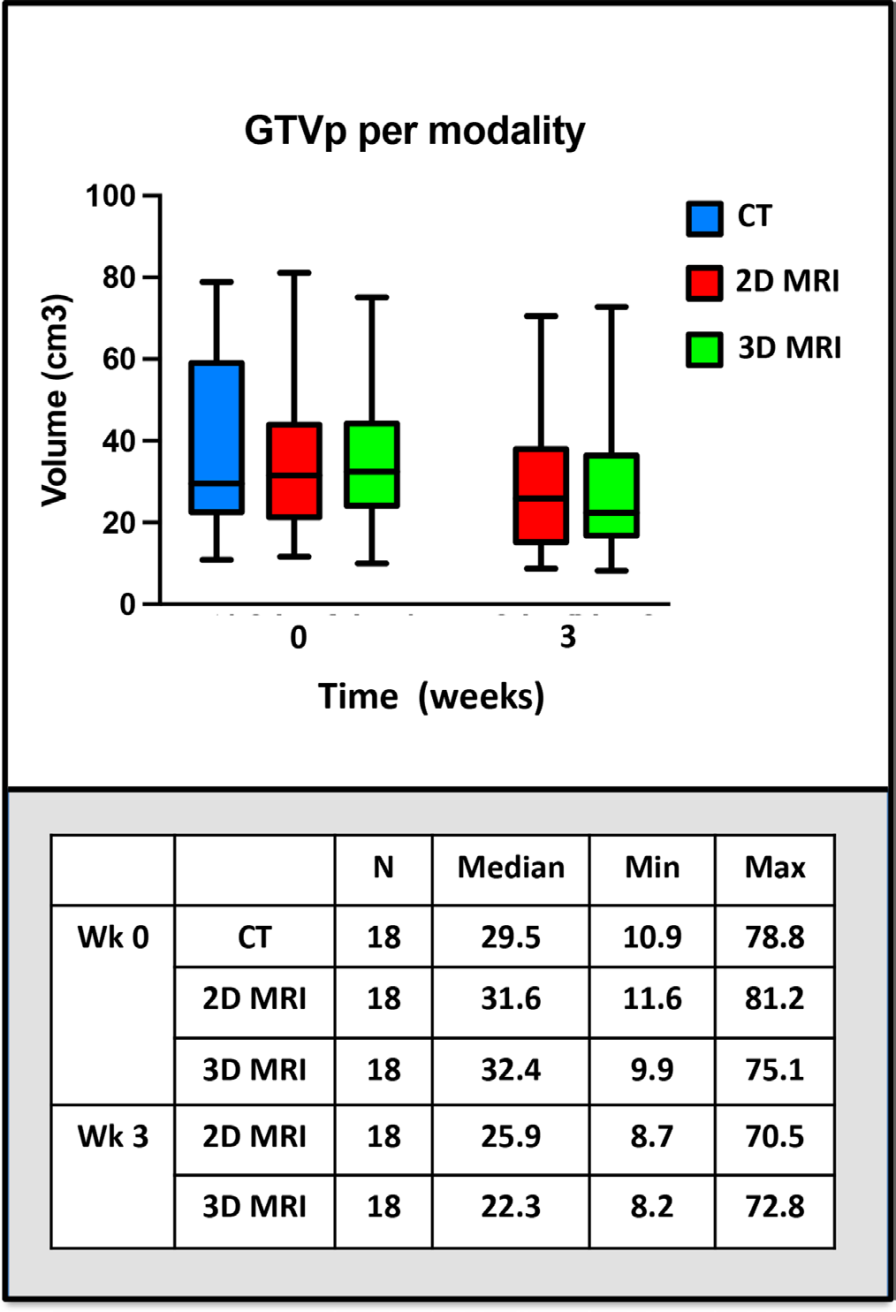
GTVp volumes per modality for all patients combined. 1. Acquired at week 0, 2. Acquired at week 3. GTVp, gross tumour volume.

Example GTVp structure delineations from all six observers on CT, 2D and 3D-MRI week 0 are shown in Figure 4. Volumes for these structures per modality for all patients combined are illustrated in Figure 5. The range of GTVp volumes for each patient were consistently smaller for MRI than CT. There was no statistically significant difference in volume between observer GTVp delineated on CT (*p* = 0.09), but there were statistically significant differences in volumes of GTVp when delineated on 2D or 3D-MRI sequences acquired week 0 and week 3; 2D-MRI week 0 (*p* = 0.01), 3D-MRI week 0 (*p* = 0.03), 2D-MRI week 3 (*p* = 0.01) and 3D-MRI week 3 (*p* < 0.01). GTVp volumes were smaller on MRI acquired at week 3 compared to MRI acquire at week 0.

### Interobserver variability

**Figure 6.**
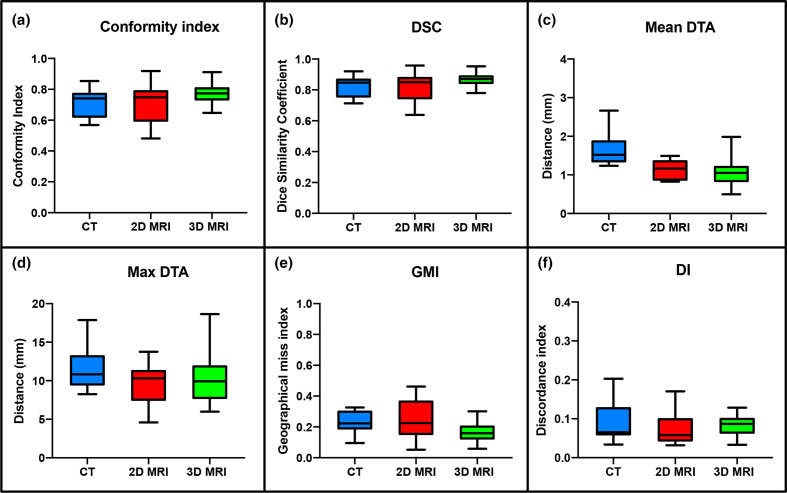
Conformity metrics for all individual CTV structures on each imaging modality at week 0. (a) CI, (b) DSC, (c) Mean DTA, (d) Maximum DTA, (e) GMI and (f) DI. CTV, clinical target volume; DI, discordance index; DSC, dice similarity coefficient; DTA, distance to agreement; GMI, geographical miss index, DI, discordance index.

Median and range IOV metric comparisons for CTV structures delineated at week 0 on the different imaging modalities are illustrated in Table 3 and Figure 6. High overlap values for CI and DSC, and low values for mean and max DTA, GMI and DI illustrate good observer agreement for CTV structures delineated on CT, 2D and 3DMRI at week 0. When comparing MRI to CT, both 2D-MRI and 3D-MRI-defined contours showed better interobserver agreement (IOA), with higher overlap values and lower distance metrics. Multiple comparison analysis showed this was statistically significant for 3D-MRI compared to CT for CI, DSC, mean DTA and GMI (*p* < 0.01). Comparison of MRI sequences showed statistically significant improvement in IOA of CTV structures on 3D-MRI for CI, DSC and GMI IOV metrics compared to 2D-MRI (*p* < 0.01).

**Table 3.** Median (range) conformity metrics for CTV structures on each imaging modality at week 0, and GTVp structures on each imaging modality at week 0 and week 3

ROI	IOV metric	Image modality		
		CT	2D-MRI	3D-MRI
Mesorectum	CI	0.83 (0.72–0.85)	0.87 (0.73–0.92)	0.89 (0.75–0.91)
	DSC	0.89 (0.83–0.92)	0.93 (0.84–0.96)	0.94 (0.85–0.95)
	Mean DTA (mm)	1.7 (1.4–2.7)	1.2 (0.8–1.5)	1.0 (0.9–2.0)
	Max DTA (mm)	11.1 (10.6–17.9)	9.8 (9.2–13.8)	9.3 (8.1–18.6)
	GMI	0.13 (0.10–0.25)	0.09 (0.05–0.24)	0.07 (0.05–0.21)
	DI	0.06 (0.03–0.06)	0.03 (0.03–0.04)	0.04 (0.03–0.05)
LN Left	CI	0.77 (0.66–0.78)	0.76 (0.66–0.79)	0.80 (0.75–0.81)
	DSC	0.87 (0.78–0.88)	0.86 (0.79–0.88)	0.88 (0.85–0.90)
	Mean DTA (mm)	1.3 (1.2–1.6)	1.3 (1.2–1.4)	1.1 (1.1–1.3)
	Max DTA (mm)	9.5 (9.4–11.1)	11.3 (8.8–12.9)	10.7 (10–12.7)
	GMI	0.19 (0.18–0.30)	0.20 (0.16–0.31)	0.14 (0.12–0.20)
	DI	0.06 (0.06–0.08)	0.06 (0.05–0.07)	0.09 (0.08–0.09)
LN Right	CI	0.76 (0.67–0.77)	0.77 (0.69–0.8)	0.79 (0.75–0.81)
	DSC	0.86 (0.80–0.87)	0.87 (0.81–0.89)	0.88 (0.86–0.89)
	Mean DTA (mm)	1.3 (1.3–1.5)	1.3 (1.1–1.4)	1.1 (1.0–1.3)
	Max DTA (mm)	11.1 (8.3–14.1)	11.0 (10.8–11.5)	11.9 (9.8–12.0)
	GMI	0.20 (0.19–0.29)	0.14 (0.20–0.28)	0.12 (0.14–0.18)
	DI	0.20 (0.19–0.29)	0.20 (0.14–0.28)	0.14 (0.12–0.18)
Presacral LN	CI	0.57 (0.56–0.60)	0.52 (0.48–0.57)	0.70 (0.64–0.72)
	DSC	0.72 (0.71–0.74)	0.68 (0.64–0.72)	0.82 (0.78–0.83)
	Mean DTA (mm)	2.0 (1.6–2.5)	0.8 (0.8–0.9)	0.7 (0.5–0.8)
	Max DTA (mm)	9.4 (9.3–15.1)	6.1 (4.6–6.9)	6.2 (6.0–7.5)
	GMI	0.32 (0.31–0.33)	0.42 (0.39–0.46)	0.23 (0.19–0.30)
	DI	0.16 (0.15–0.20)	0.17 (0.11–0.17)	0.13 (0.10–0.13)
GTVp week 0	CI	0.58 (0.56–0.61)	0.61 (0.52–0.77)	0.69 (0.54–0.81)
	DSC	0.72 (0.65–0.73)	0.76 (0.66–0.87)	0.81 (0.67–0.89)
	Mean DTA (mm)	1.9 (1.2–2.3)	1.2 (1.0–2.0)	1.6 (1.3–2.1)
	Max DTA (mm)	11.5 (10.1–17.7)	8.4 (8.4–10.8)	11.1 (9.9–11.6)
	GMI	0.36 (0.36–0.41)	0.33 (0.20–0.42)	0.26 (0.09–0.40)
	DI	0.05 (0.05–0.09)	0.06 (0.04–0.07)	0.08 (0.07–0.11)
GTVp week 3	CI		0.56 (0.46–0.61)	0.6 (0.51–0.73)
	DSC		0.70 (0.60–0.74)	0.70 (0.64–0.83)
	Mean DTA (mm)		2.0 (1.9–2.0)	2.0 (1.8–2.3)
	Max DTA (mm)		9.4 (9.3–11.3)	10.0 (9.8–12.9)
	GMI		0.38 (0.32–0.46)	0.37 (0.15–0.41)
	DI		0.08 (0.06–0.10)	0.10 (0.08–0.13)

CTV, clinical target volume; DI, discordance index; DSC, dice similarity coefficient; DTA, distance to agreement; GMI, geographical miss index; GTVp, gross tumour volume; IOV, interobserver variability; LN, lymph node.

**Figure 7.**
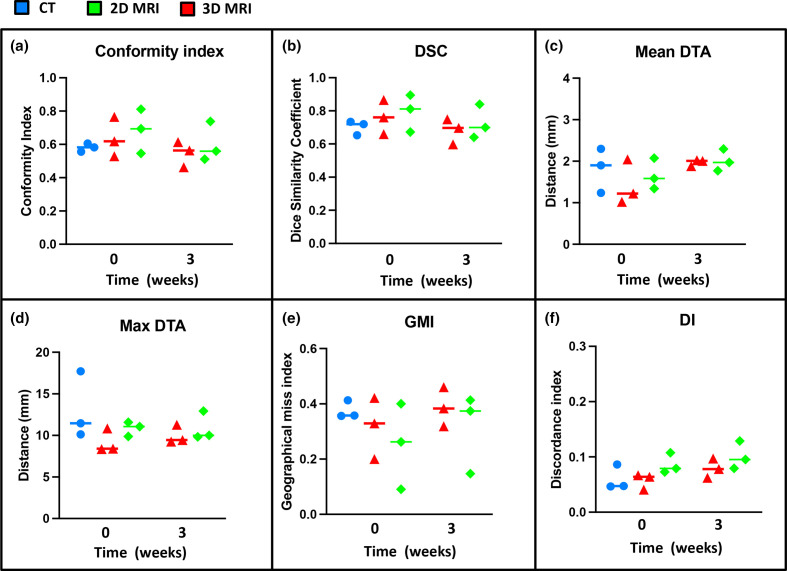
Conformity metrics for GTVp on each imaging modality at week 0 and week 3. (a) CI, (b) DSC, (c) Mean DTA, (d) Maximum DTA, (e) GMI and (f) DI. DI, discordance index; DSC, dice similarity coefficient; DTA, distance to agreement; GMI, geographical miss index; GTV, gross tumour volume

Median and range IOV metric comparisons for GTVp structures delineated week 0 and week 3 on different imaging modalities are illustrated in Table 3 and in Figure 7. On comparison between MRI and CT-defined GTVp contours at week 0, both 2D-MRI and 3D-MRI showed consistently better IOA with the STAPLE gold standard contours across all IOV metrics, with 3D-MRI performing better than 2D-MRI. This did not however reach statistical significance for most IOV metrics. Better IOA in MRI-defined GTVp contours was seen at week 0 compared to week 3 across all IOV metrics and this reached statistical significance for CI, DSC and GMI (*p* < 0.05). IOA was lower for GTVp than for CTV structures delineated on week 0 imaging across all modalities.

### Topographic analysis

**Figure 8.**
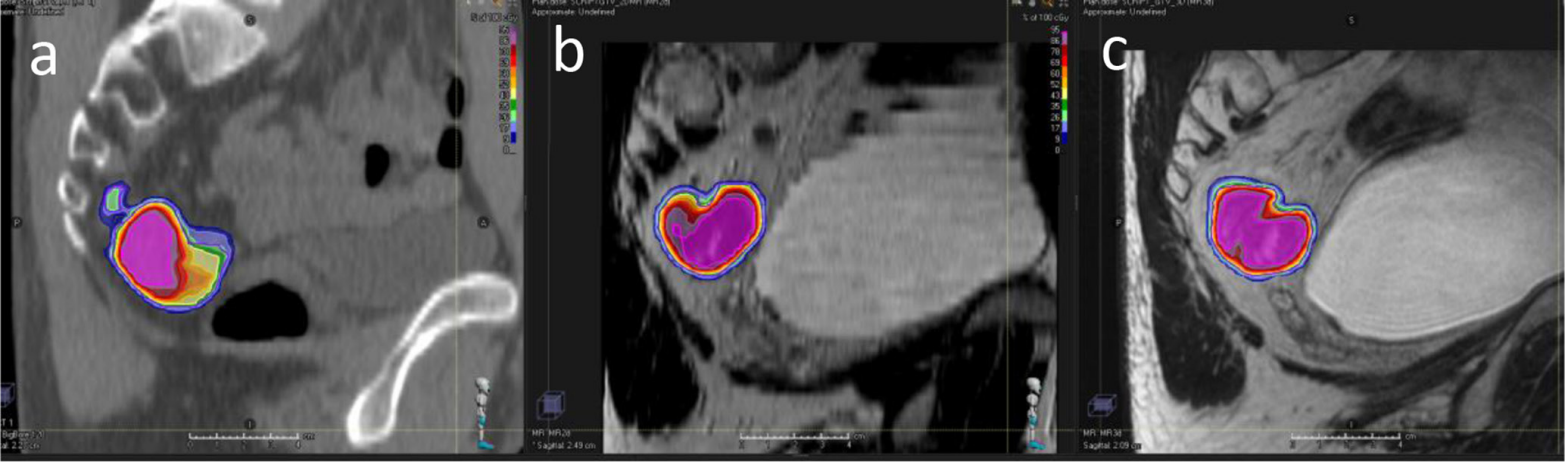
Consensus agreement map for GTVp. (a) CT, (b) 2D-MRI and (c) 3D-MRI. Pink represents 95% agreement between observers in the volume to be contoured in the delineated structure. GTVp, gross tumour volume.

**Figure 9.**
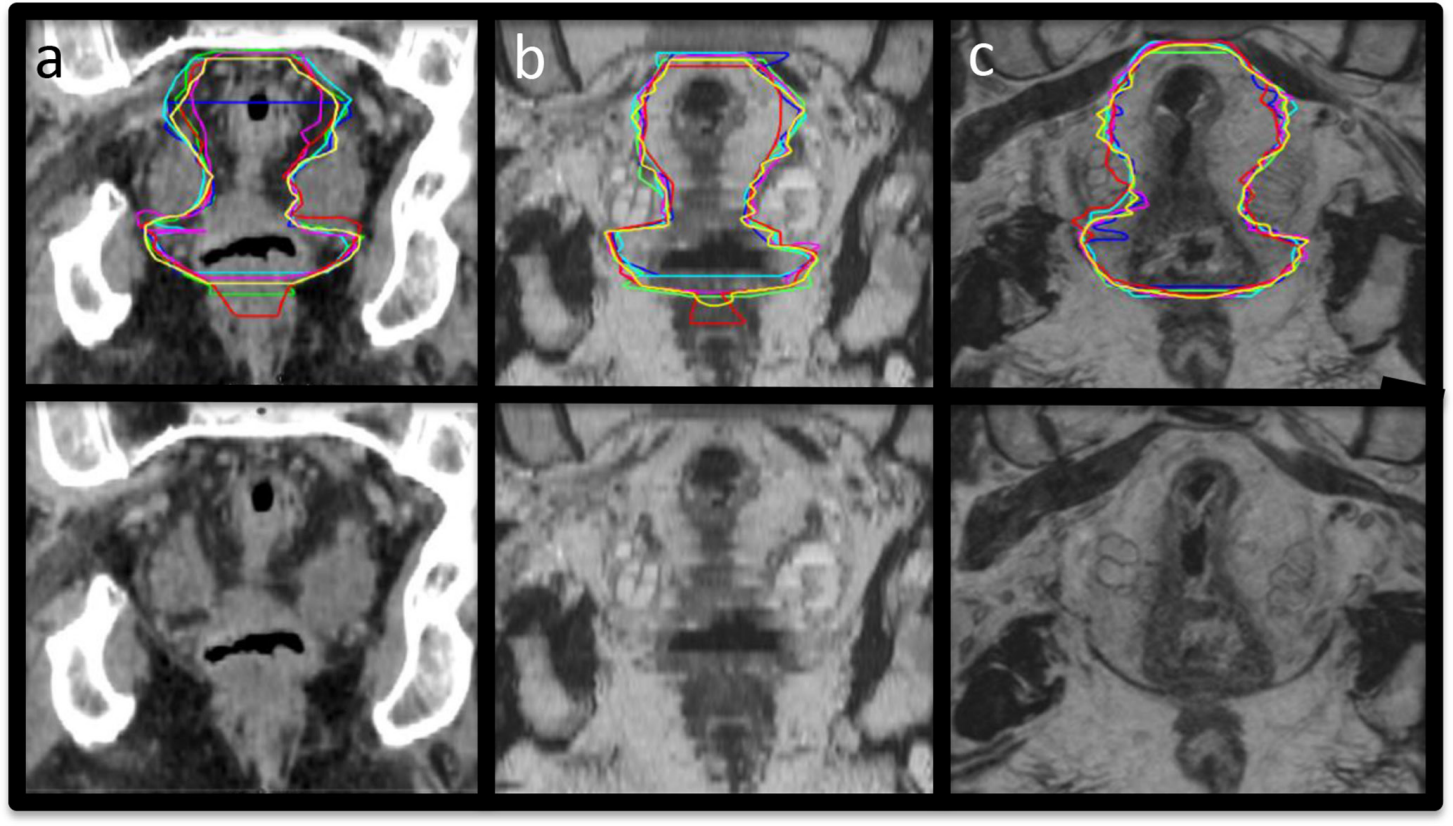
Coronal images of mesorectum with and without observer contours. (a) CT and (b) 2D-MRI and (c) 3D-MRI.

Topographic analysis using consensus agreement maps demonstrated that contour heterogeneity across all imaging modalities was greatest for GTVp, and this was worse for contours defined on week 3 imaging compared to week 0. Variability between observers was largest at superior/inferior GTVp extent. The largest variability was seen on CT, and improved on 2D-MRI, and was best on 3D-MRI. This is illustrated in Figure 8 where 100% agreement between observers in the volume to be included in the GTVp occurs at largest percentage volume for 3D-MRI. On week 0 imaging, the largest variability in CTV delineation was seen at the superior/inferior extent of the mesorectum (Figure 9). This variability was less on 3D-MRI. Other areas of contour heterogeneity were: superior/inferior extent of the LN regions, anterior extent of the presacral LN volume, and lateral extent of the right and left LN volumes in the obturator region.

## Discussion

To our knowledge, this is the first study that has evaluated IOV in rectal radiotherapy CTV delineation on MRI, or directly compared spatial IOV in GTVp defined on CT with MRI. This is also the first study that has evaluated IOV in target volume delineation on MRI suitable for RTP, and IOV in GTVp delineation on MRI acquired during radiotherapy treatment course. This study demonstrates excellent IOA for CTV contours delineated on 3D-MRI sequences at week 0 (CI ≥0.7, DSC ≥ 0.82). Agreement in CTV delineation is better on MRI-based images compared to CT and agreement in CTV delineation is better on 3D-MRI compared to 2D-MRI. Agreement of MRI defined GTVp week 0 is good, and superior to that on CT. MRI defined GTVp contour agreement week 3 of radiotherapy is worse when compared to MRI acquired week 0.

Across all CTV structures, observer contour agreement was better on MRI compared to CT, with 3D-MRI performing better than 2D-MRI (median CI 0.74 for CT, 0.75 for 2D-MRI and 0.77 for 3D-MRI). The IOV data for CT defined CTV in this study are comparable to work by Fuller et al^18^ who measured IOV on CT of “CTVA”, consisting of the pelvic LNs and mesorectum. Although the absolute differences in CI across CT, 2D and 3D-MRI in our study were small, in some cases we expect this would have significantly impacted on the dose received. For the mesorectum, the largest differences were seen inferiorly, which defines the inferior extent of the CTV in most patients. Maximum distance to agreement of individual observer contours and the STAPLE contour in individual patients were 17 mm for CT, 12–14 mm for 2D-MRI and 10–12 mm for 3D-MRI. With increasingly conformal treatments differences of 5 mm will result in clinically significant dosimetric differences, which could affect clinical outcome.

MRI defined GTVp week 0 had better agreement for GTVp across all IOV metrics compared to CT. The CI was 0.58 for CT, 0.62 for 2D-MRI and 0.7 for 3D-MRI. This is comparable to published data.^19,20^ Burbach et al^19^ looked at GTVp contour agreement on 2D *T*_2_W and diffusion-weighted imaging (DWI) MRI following consensus agreement and training for GTVp delineation. They found that CI for GTVp defined on 2D *T*_2_W MRI at 0.7, was better than CI for 2D *T*_2_W MRI-defined GTVp in our study. This is expected, as there was no use of GTVp delineation guidelines in our study. In our study however, the max DTA values for GTVp across all imaging modalities, including CT, were smaller; max DTA 18 mm for CT, 11 mm for 2D-MRI and 12 mm for 3D-MRI. This compared to Burbach et al; max DTA 31 mm for 2D-MRI and 49 mm for DWI.

In our study, across all imaging modalities, the most frequent and largest variability in CTV contours were seen at the mesorectum superior/inferior extent, presacral LNs anterior extent, and lateral extent of the right and left LNs in the obturator region. These sites of largest variability occurred at the edge of the composite volume of all the individual CTV structures. This constitutes the combined CTV, and this variability therefore has potential to impact on treatment.

Variability in the superior extent of the mesorectum was likely due to ambiguity in interpretation of guidelines. The level where the internal iliac artery bifurcates is hard to identify because the bifurcation occurs over multiple axial slices. The inferior border of the mesorectum, defined as the point of levator ani muscle insertion, is best seen on sagittal view. This was difficult to see on CT. It was also difficult to see on 2D-MRI, because these sequences, which were acquired axially, do not reconstruct well in sagittal view. This is in contrast to the isotropic voxel 3D-MRI, which reformats well into any orientation. The benefit of defining the inferior extent of mesorectum on 3D-MRI is clearly seen in Figure 9. Contour variability at the anterior border of the presacral LNs was largest for CT and 2D-MRI, where slice intervals of 2.5 mm and 3 mm respectively, led to partial volume effect. Another common area of IOV was the lateral extent of the right and left LNs in the obturator region. This is most likely due to ambiguity in interpreting the guidelines as to what constitutes the ‘fat area posterior to the ilium’. Improved training and peer review would help reduce variability at the lateral extent of the right and left LNs, but for the inferior extent of the mesorectum and the anterior extent of the presarcal LNs we have reached a limit to what can be achieved with CT and 2D axial MRI, and 3D-MRI or 2D sagittal imaging is required.

Variability in GTVp was seen throughout the volume at week 0. Observers were asked to contour visible tumour, omitting the lumen and uninvolved rectal wall, and were asked not to include the whole rectal circumference. On CT, it is not possible to differentiate tumour from normal rectal wall, because image contrast in these structures is identical. On *T*_2_W MRI GTVp is easier to define, because tumour returns intermediate signal intensity in contrast to normal rectal mucosa and submucosa high signal, and muscularis propria low signal. Uncertainty in GTVp delineation must be accounted for in rectal boost studies to ensure adequate target coverage.

IOV in GTVp was worse at week 3 compared to week 0. During radiotherapy treatment course, inflammation within tumour and normal tissue result in high signal intensity oedema and subsequent low signal intensity fibrosis. It is therefore difficult to distinguish viable cancer from treatment-induced fibrosis during and after radiotherapy. There are no published data on how to interpret MRI for GTVp assessment during radiotherapy. There is high agreement in rectal cancer staging between diagnostic high resolution *T*_2_W MRI and pathology,^21,22^ but interpretation following CRT (chemoradiotherapy) is difficult.^23^ Addition of DWI is reported to help differentiate between scar tissue and residual tumour.^24^ But DWI cannot be used alone as it does not provide anatomical information.

There are no published guidelines on how to interpret MRI during radiotherapy. Standardisation of GTVp delineation on MRI during treatment is essential in the context of MRI-guided ART and dose escalation. During RTP, there is time to acquire multiple MRI sequences to aid target delineation, but during the online MRI-guided ART workflow a single MRI sequence is used for target and OAR delineation and re-optimisation of the treatment plan based on target geometry at the time of treatment. This sequence must be 3D, provide anatomical information and an external body contour, and be geometrically accurate. A 3D *T*_2_W sequence is therefore preferable. Consensus agreement on what constitutes GTVp on MRI needs to be agreed and there is an urgent requirement for formal training in MRI interpretation for radiation oncologists. This is necessary for robust and reproducible contouring within future clinical trials designed to investigate MRI-guided ART for rectal boost.

Strengths of this work are that it is the first study to evaluate and compare IOV in CT and MRI defined CTV and GTVp using a true GTVp definition; gross visible tumour, excluding normal rectal wall. It is the first study to evaluate CTV and GTVp delineation IOV on 3D-MRI sequences suitable for RTP and compare this against 2D-MRI. And, it is the first study to look at MRI-defined GTVp on images acquired during treatment course. This work has highlighted the need for consensus agreement and guidelines in GTVp delineation on MRI before and during radiotherapy treatment course.

The biggest limitation in our study is lack of training in MRI interpretation for GTVp definition both before and during radiotherapy treatment. With guidelines, IOV in GTVp delineation should improve. Observers in this study, all from the same institution, had between 5 and 15 years’ experience of CT based contouring for rectal radiotherapy, but limited contouring experience on MRI. They have been considered experts in CT, but not MR-based target delineation. Despite limited experience in delineation on MRI, excellent agreement in MRI-defined CTV and good agreement in MRI-defined GTVp was observed. Additional improvement in the agreement between observers is expected with further training and increased experience in MRI interpretation, particularly with MRI acquired during treatment. No correlation for IOV and clinical expertise and experience was performed.

In conclusion, this study has found excellent IOA in CTV delineation and good IOA in GTVp delineation at week 0 in MRI sequences suitable for RTP. IOA in target volume delineation was better of MRI-based RTP images compared to CT at week 0 and IOA for target volume delineation was better on 3D-MRI compared to 2D-MRI. IOA for rectal GTVp delineation was worse at week 3 compared to week 0, on 2D and 3D-MRI sequences. This work supports the use of MRI sequences designed and customised for CTV and GTVp definition and confirms that 3D-MRI, which is necessary for RTP, produces best agreement in both CTV and GTVp delineation. Consensus guidelines for GTVp delineation on MRI acquired before and during treatment will further improve observer agreement and are necessary in the context of dose escalation MRI-guided ART rectal boost studies.
